# Assessment of Targeted and Non-Targeted Responses in Cells Deficient in *ATM* Function following Exposure to Low and High Dose X-Rays

**DOI:** 10.1371/journal.pone.0093211

**Published:** 2014-03-28

**Authors:** Anne Kiuru, Meerit Kämäräinen, Sirpa Heinävaara, Katri Pylkäs, Kim Chapman, Armi Koivistoinen, Teuvo Parviainen, Robert Winqvist, Munira Kadhim, Virpi Launonen, Carita Lindholm

**Affiliations:** 1 STUK-Radiation and Nuclear Safety Authority, Helsinki, Finland; 2 Laboratory of Cancer Genetics and Tumor Biology, Department of Clinical Chemistry and Biocenter Oulu, University of Oulu, Oulu University Hospital, Oulu, Finland; 3 Brookes University, Oxford, United Kingdom; National Research Council, Italy

## Abstract

Radiation sensitivity at low and high dose exposure to X-rays was investigated by means of chromosomal aberration (CA) analysis in heterozygous *ATM* mutation carrier and A-T patient (biallelic *ATM* mutation) lymphoblastoid cell lines (LCLs). Targeted and non-targeted responses to acutely delivered irradiation were examined by applying a co-culture system that enables study of both directly irradiated cells and medium-mediated bystander effects in the same experimental setting. No indication of radiation hypersensitivity was observed at doses of 0.01 Gy or 0.1 Gy for the *ATM* mutation carrier LCL. The A-T patient cells also did not show low-dose response. There was significant increase in unstable CA yields for both *ATM* mutation carrier and A-T LCLs at 1 and 2 Gy, the A-T cells displaying more distinct dose dependency. Both chromosome and chromatid type aberrations were induced at an increased rate in the irradiated A-T cells, whereas for *ATM* carrier cells, only unstable chromosomal aberrations were increased above the level observed in the wild type cell line. No bystander effect could be demonstrated in any of the cell lines or doses applied. Characteristics typical for the A-T cell line were detected, i.e., high baseline frequency of CA that increased with dose. In addition, dose-dependent loss of cell viability was observed. In conclusion, CA analysis did not demonstrate low-dose (≤100 mGy) radiosensitivity in *ATM* mutation carrier cells or A-T patient cells. However, both cell lines showed increased radiosensitivity at high dose exposure.

## Introduction

Uncertainty in estimating health risk of low dose or low dose rate exposure to ionizing radiation (IR) is to a large extent caused by the absence of epidemiological evidence [1] but also on the insufficient knowledge of cellular mechanisms [2], [3] on which such assessment could be based on. A multitude of processes may be activated after low dose radiation exposure, including inflammatory and cytokine signalling as well as DNA damage response. There is evidence that individuals with impaired DNA damage control have an increased risk of carcinogenesis at low dose exposure [4]. In addition to the DNA targeted response of low dose radiation, observations on non-targeted responses such as bystander effects, genomic instability and adaptive response have been reported [5]. It has been suggested that at very low doses, the majority of cellular responses belong to the bystander effect [6]. In radiation induced bystander effect, the non-irradiated cells elicit biological responses after communication with irradiated cells via gap junctions or soluble factors secreted by irradiated cells. The effect has been studied in different cell types and radiation qualities using endpoints like DNA damage, chromosomal aberrations, cell death, changes in gene expression, and epigenetic changes [6], [7].

Comprehension of low dose response, whether targeted or non-targeted, is further complicated by heterogeneity of radiation response among individuals. This variability is often based on genetic predisposition and it is known that certain genetic disorders display increased sensitivity to radiation. The rare autosomal recessive ataxia-telangiectasia (A-T) [MIM#208900] is one such disorder characterized by chromosomal instability, neurological degeneration, immune dysfunction, and high cancer incidence. The A-T phenotype is caused by mutations in the *ataxia-telangiectasia mutated* gene (*ATM)* [MIM*607585] [8].

At the cellular level, A-T patients are known to be extremely radiosensitive and they show chromosomal instability due to defects in double-strand break (DSB) repair and multiple defects in signaling pathways including G1/S, S, and G2/M cell cycle checkpoints, apoptosis, and chromatin remodeling (reviewed in [9]). Decreased cell survival of the A-T cells after exposure to radiation has been reported [10]–[13]. A-T cells show increased yields of chromosomal aberrations (CAs) after high dose (>1 Gy) of ionizing radiation with X- or γ-rays. However, less is known about how these cells respond to low dose exposures i.e. levels less than about 100 mGy low LET radiation, that are important in radiation protection of the general population as well as for understanding the mechanisms of radiation effects. The low dose response of A-T and other repair deficient cells has been shown to diverge from effects observed at high dose. Considering the yield of exchange type aberrations, increased levels of aberrations were observed at high dose for A-T cells, whereas at low dose range of 0.5 Gy and below, the frequencies were similar to wild type cells [10], [14]. It was thus concluded that the radiation sensitivity of *ATM* deficient cells occurs at high but not at low doses. Regarding heterozygous *ATM* mutation carriers, the radiation sensitivity of the cells is either overlapping with that of normal cells or is intermediate between normal and A-T cells [15], [16]. *ATM* mutation carriers are estimated to present up to 1% of the normal population and there are indications of an increased risk of breast cancer among this group [8], [12], [17]–[19].

In this study, we compared low dose (0.01 and 0.1 Gy X-ray) and high dose (1 and 2 Gy) radiosensitivity assessed by induced chromosomal aberrations and viability assay. A co-culture system was used which enabled the evaluation of responses concurrently in directly irradiated cells as well as medium mediated bystander effects. Two radiation-sensitive *ATM* lymphoblastoid cell lines were studied; one from a breast cancer patient carrying an *ATM* mutation and one from an A-T patient. In addition, an *ATM* wild type cell line was included as control. The aim was to evaluate the influence of genetically determined radiosensitivity in targeted and bystander cells.

## Materials and Methods

### Ethics statement

Ethics approval and consent procedure of the entire study were obtained from the Ethical Board of the Northern Ostrobothnia Health Care District and the Finnish Ministry of Social Affairs and Health. Cell lines BR0409 and BR0996 exploited in the present study were anonymous and coded, and therefore personal identification was not possible. Moreover, cell line from AT-patient (GM03332) (A-T LCL) carrying a homozygous truncating mutation 7913 G>A in the *ATM* gene, and purchased from the Coriell Institute for Medical Research (Camden, New Jersey, USA), was delivered without personal data.

### Cell culture

The study was conducted using three Epstein-Barr virus (EBV)-immortalized lymphoblastoid cell lines (LCLs) derived from: 1) a healthy individual, 2) a breast cancer patient, and 3) an A-T patient. Lymphoblast cell lines were established and screening for ATM germline mutations were performed in an earlier study for the healthy individual (BR0409) and the breast cancer patient (BR0996) [12]. No mutations were detected in the healthy individual (WT LCL). A heterozygous truncating 6903insA mutation was found in the breast cancer patient (*ATM* carrier LCL) and the lymphoblast cell line from this patient was established at the age of 40, four years after the diagnosis. BR0409 derived from a healthy non-carrier (age 54) from the same family as BR0996. Both LCLs were created with Epstein-Barr virus (B95-8) transformation (Oulu University Hospital, Oulu, Finland).

All cell lines were cultured in suspension in RPMI 1640 medium (Lonza, Verviers, Belgium) supplemented with 15% (v/v) of fetal bovine serum (Gibco Invitrogen, Paisley, UK), L-glutamine (2 mM) (Gibco Invitrogen, Paisley, UK), and streptomycin-penicillin (100 µg/ml and 100 units/ml, respectively; Gibco Invitrogen, Paisley, UK). Cell population densities were determined using an automated cell counter (Invitrogen, Carlsbad, CA, USA).

### Co-culture assay and irradiations

**Figure 1:**
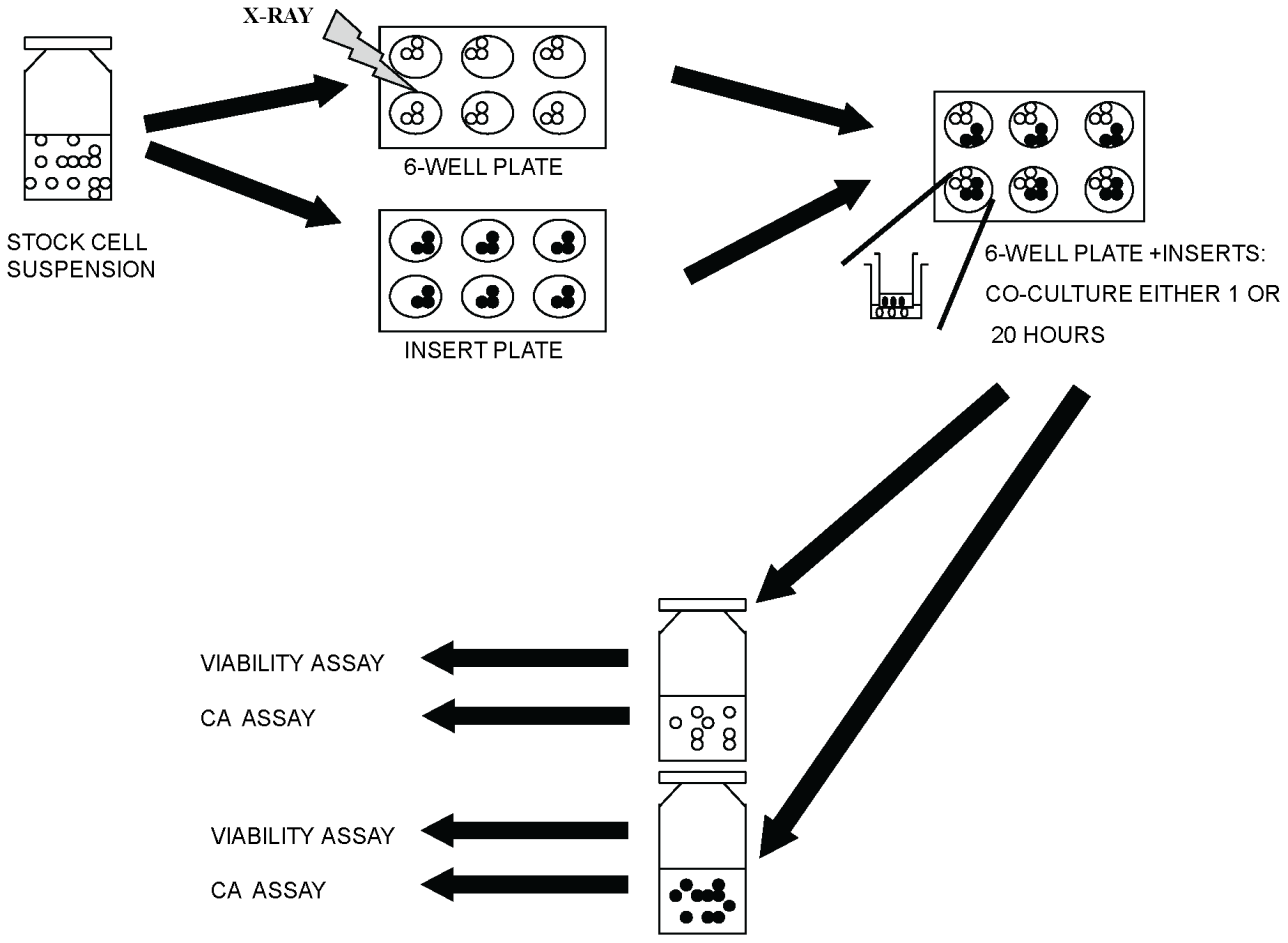
Overview of the experimental design used in the co-culture assay. In each experiment, LCLs of the same genotype were seeded into 6-well plates (cells exposed to direct irradiation) and insert plates (bystander cells). Cells in 6-well plates (indicated by white circles) were irradiated with X-ray at doses of 0, 0.01, 0.1, 1, or 2 Gy. Immediately after irradiation, inserts with a permeable filter and containing bystander cells (filled black circles) were placed on top of the cells in the 6-well plates. After 1 or 20 hours co-culture, the directly irradiated and bystander cells were separated and incubated for an additional 24 hours before CA assay and 43 hours before cell viability assay were performed. Total post-irradiation time was 44 or 63 hours in the cell viability assay and 25 or 44 hours in the CA assay.

Cells destined to be irradiated or bystander affected by diffusible factors were seeded at a cell density of 2.5×10^5^/ml into 6-well plates (Falcon, Becton Dickinson Labware, Franklin lakes, NJ, USA) or cell culture inserts (pore size 3.0 µm, pore density 2.0±0.2×10^5^/cm^2^, Falcon, Becton Dickinson Labware, Franklin lakes, NJ, USA), respectively, with 2 ml volume in each well or insert. Overview of the co-culture method is shown in Figure 1. The cells in 6-well plates were irradiated with at 0.01, 0.1, 1, or 2 Gy of X-rays (100 kV, wide range energy spectrum, CPI Indigo 100, Communication and Power Industries). Dose rates were adjusted according to dose: 0.02–0.06 Gy/min for 0.01 and 0.1 Gy, and 0.22–0.25 Gy/min for 1 and 2 Gy. Control cells were treated in a similar manner as the irradiated cells, except for exposure. Immediately after irradiations inserts containing the corresponding cell line were placed on top of the 6-well plates and the cells were co-cultured for either 1 or 20 hours. After co-culture, cells from one 6-well plate were collected into T25 culture flasks. Similarly, cells from the six co-culture inserts were pooled into flasks. All experiments were performed three times.

### Chromosomal aberration (CA) analysis

For chromosomal aberration (CA) analysis, to allow proliferation into next cell division, the cells pooled into T25 culture flasks were incubated for another 24 hours, i.e., total culture time of 25 or 44 hours post-irradiation, including Colcemid treatment to a final concentration of 20 ng/ml for 1.5 hours. For harvesting, the cells were centrifuged and treated with hypotonic solution (0.075 M KCl) at room temperature for 20 minutes and fixed with methanol∶acetic acid (3∶1). Metaphase spreads were prepared on moist slides, dried and stained with 4% Giemsa.

Metaphases were located using an automated metaphase finder (Metafer, Metasystems, Altlussheim, Germany). CA analyses were performed by two scorers on 50 metaphases each from each of the three replicates of the experiments with WT and *ATM* carrier cells, resulting in 300 cells in total per data point. Since the basal level of chromosomal aberrations in A-T LCL was higher than the level in the *ATM* carrier and WT cell lines, a smaller number of analysed cells were required, i.e. 175 metaphases per data point. Scoring was performed on blind coded slides. Both chromosome- and chromatid-type aberrations were scored. The chromosome-type aberrations included dicentric chromosomes with or without an acentric fragment, ring chromosomes with or without a centromere, acentric fragments (also double minutes), chromosome breaks, and marker chromosomes. The chromatid-type aberrations contained chromatid breaks and fragments, as well as exchanges.

### Cell viability test

From each T25 flask, containing pooled cells from either irradiated or bystander wells, 10^4^ cells were seeded into a 96-well plate (6 parallels). In order to detect differences between cell proliferation rates, cells were cultured for additional 43 hours, i.e., in total 44 or 63 hours post-irradiation culture. Cell proliferation rate of different groups was determined by using the cell proliferation kit I (MTT, 3-[4,5-dimethylthiazol-2-yl]-2,5-diphenyl tetrazolium bromide) (Roche Diagnostics, Mannheim, Germany) according to the instructions of the manufacturer. The dissolved formazan dye was spectrophotometrically measured using an ELISA reader (Benchmark Microplate Reader, BioRad, CA, USA) with wavelength of 595 nm. The absorbance value correlates directly to the number of viable cells.

### Statistical analyses

The viability and chromosomal data were analyzed separately by the type of exposure setting (directly irradiated or bystander cells) and time points. For the analyses of chromosomal data, chromosomal aberrations were divided in two categories: unstable CAs (dicentric chromosomes, fragments, chromosome breaks, and rings) and chromatid-type aberrations (fragments, chromatid breaks, and exchanges). The aberrations were also summed over all the experiments by the exposure setting, dose, time point and cell line. The effect of dose on the aberration frequency was visualized with dose-response curves. In the dose-response analyses, dose was applied as linear and quadratic, if needed and adjusting for overdispersion (i.e., with variance to mean ratio) arising from original observations. The frequency of chromosomal aberrations were compared between the LCLs (WT vs. carrier and WT vs. A-T) by the type of exposure setting (directly irradiated or bystander cells), time point post irradiation and dose using z-test for comparing two Poisson-distributed counts. The aberration frequency was also compared between the doses (0 Gy vs. 0.01 Gy, 0.1 Gy, 1 Gy or 2 Gy) by the type of exposure setting, time point and LCL using the same test. Due to multiple comparisons, p-values were corrected with the Holm-Bonferroni correction (between LCLs and doses, respectively).

The viability with the logarithms of individual viability measurements (i.e., ln(viability)) were analyzed with a mixed model. The dose as numerical and LCL were included in a model as fixed (covariate) effects, and experiment and plate as random effects. The logarithmic transformation was done to improve the goodness of a model. The first-order interactions were included into a model and considered statistically significant at 10% level. In the primary analyses all doses were included into the model. If the effect of dose was significant, secondary analyses were done by restricting the data to the low doses of radiation (0, 0.01 and 0.1 Gy). Outlying and extreme observations, 2–3% of the data, were excluded from the reported models. The exclusion of these observations had no effect on conclusions and only a slight effect on reported estimates.

All tests were performed two-sided with 5% level significant unless stated otherwise. Data were analyzed with Stata/IC version 12 except the dose response analyses which were performed with R statistical programming environment.

## Results

### Chromosomal aberration (CA) analysis

**Figure 2:**
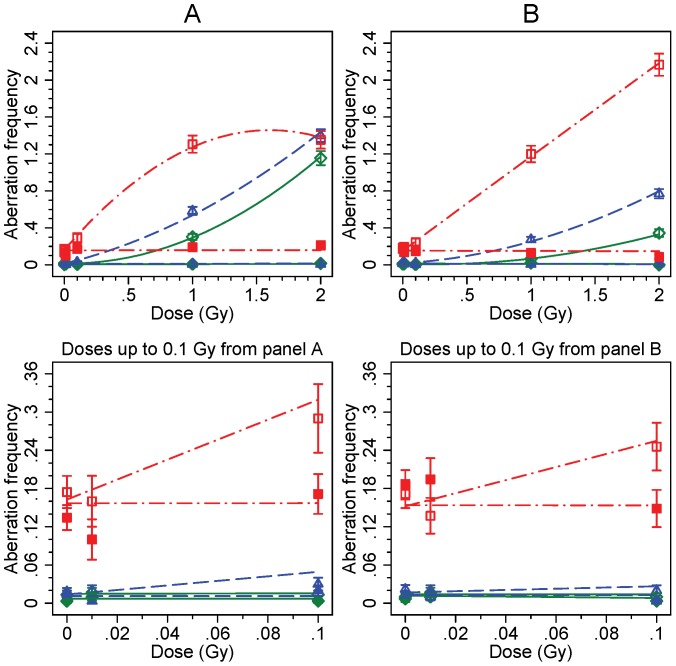
Frequencies of unstable chromosome-type aberrations. Number of aberrations per 100 cells is shown for direct exposure (open symbols) to X-ray irradiation and for bystander cells (filled symbols). Observed aberration rates pooled from parallel experiments and fitted curves are presented. The WT LCL is represented by (◊) and solid lines (—) in green, the *ATM* carrier LCL by (Δ) and dashed lines (– –) in blue, and the A-T LCL by (□) dash/dot lines (– · –) in red, all at both 25 hours (including 1 h co-culture; panel A) and 44 hours (including 20 h co-culture; panel B) post-irradiation. Results for low doses are shown in the lower panels. Observations are reported with Poisson distributed errors bars (± S.E.).

**Figure 3:**
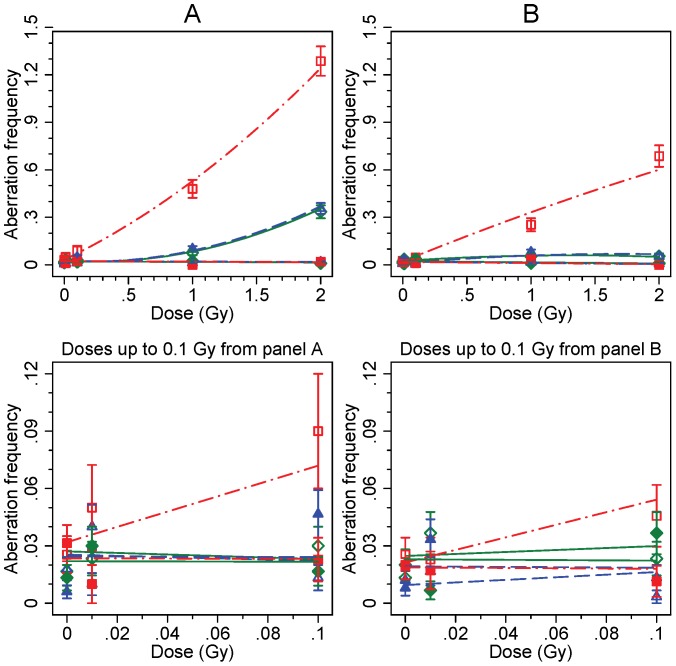
Frequencies of chromatid-type aberrations. See Figure 2 legend for details.

Results from dose-response analyses of chromosomal aberrations (CA) are shown in Figures 2 and 3, for unstable chromosome-type and for chromatid-type aberrations, respectively, containing results of both irradiated and bystander cells. In cells directly irradiated with up to 2 Gy dose, unstable chromosomal aberrations, consisting about equally of both chromosome fragments and dicentric chromosomes, increased with linear-quadratic dose in all studied LCLs both at 25 and at 44 hours post-irradiation (Figure 2). The highest increase was observed among the A-T cells, as expected. However, at 25 hours and 2 Gy dose, an apparent saturation of unstable aberration yields was detected. Compared with the WT cells, increased unstable CA yields were also induced in the *ATM* carrier cells, both at 25 and 44 hours. At 44 hours, however, the WT and the *ATM* carrier LCLs irradiated with doses of 1 and 2 Gy displayed lower frequencies of cells with unstable aberrations as compared to 25 hours. Such trend was not observed for the A-T cells, a consequence of inability of the *ATM* mutated cells to deal with radiation induced damage.

The lower panel in Figure 2 indicates that the baseline frequency of CAs in the mutant cell line is more than ten times higher set against the *ATM* carrier and wild type cell lines. Further, the figure demonstrates that irradiation at small doses, i.e. up to 100 mGy, resulted in almost a doubling of aberration frequencies for the A-T cell, whereas a very small increase was observed for the *ATM* carrier LCL at 25 hours. The rates of unstable aberrations of the bystander cells are also shown in Figure 2. Aberrations observed in bystander cells were not increased with dose but remained at the control level for all dose and time points.

Frequencies of chromatid-type aberrations differed in many aspects from the unstable CAs (Figure 3). The baseline frequency of chromatid-type aberrations was equal in all cell lines. Secondly, A-T LCL showed distinct dose-dependent increase at both 25 and 44 hours, whereas the carrier and WT cell lines showed only modest elevation of chromatid aberrations. Moreover, it is apparent that chromatid-type aberrations observed at 25 hours are to a large extent expressed one cell cycle later as chromosomal aberrations among the 44 hour data. Chromatid-type aberrations in bystander cells, as demonstrated in Figure 3, were not affected and remained generally at baseline level at both 25 and 44 hours. The data collected from all CA analyses are presented in Table S1.

The Z-tests for Poisson distributed counts confirmed generally the observations made on the basis of Figures 2 and 3 (data not shown). Particularly, the yield of unstable CAs in irradiated cells in the A-T LCL was significantly increased compared with WT LCL at nearly all dose points at 25 and 44 hours, except at 2 Gy dose at 25 hours. CAs in bystander A-T cells also showed significant increase set against WT cells, but this was entirely due to the high baseline level of the A-T cell line. Furthermore, in comparison to the WT LCL, a significant increase in unstable CA yields was observed for the *ATM* carrier LCL at high doses (1 and 2 Gy) for both time points. On the other hand, chromatid-type aberrations were significantly elevated only in the A-T cells at high doses. This observation indicates that chromatid-type aberrations do not occur spontaneously in the A-T LCL, but these are induced at the G2-phase in the proliferating cells. Additionally, pair wise Z-tests for each dose performed against 0 Gy showed that unstable aberration frequencies were significantly increased at high doses (1 and 2 Gy) in the *ATM* carrier LCL and A-T LCL, whereas the yield of chromatid aberrations increased at high doses for the A-T LCL only. The Z-test revealed no significant differences in the bystander cells, as also seen in the figures.

### Cell viability test

**Figure 4:**
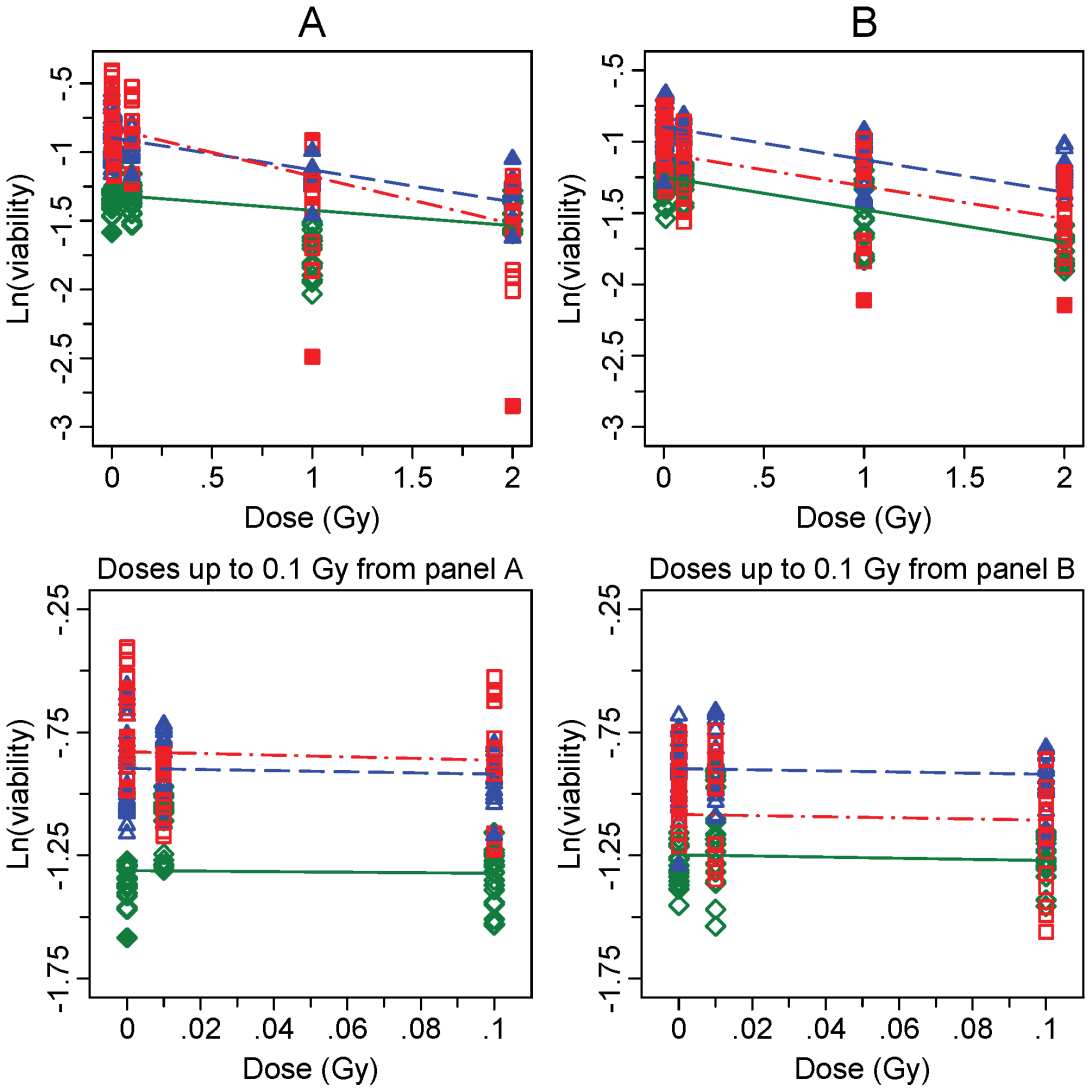
Cell viability after direct exposure. Measured colorimetric values and fitted lines determined by the viability assay after X-ray irradiation are presented in logarithmic scale. The WT LCL is represented by (◊) and solid lines (—) in green, the *ATM* carrier LCL by (Δ) and dashed lines (– –) in blue, and the A-T LCL by (□) dash/dot lines (– · –) in red, all at both 44 hours (including 1 h co-culture; panel A) and 63 hours (including 20 h co-culture; panel B). Outlying observations within experiments excluded from underlying models are presented with the respective solid symbols i.e., with diamond, triangle and square for the WT LCL, the *ATM* carrier LCL and the A-T LCL, respectively. An individual symbol represents the data from one measurement. Results for low doses are shown in the lower panels.

**Figure 5:**
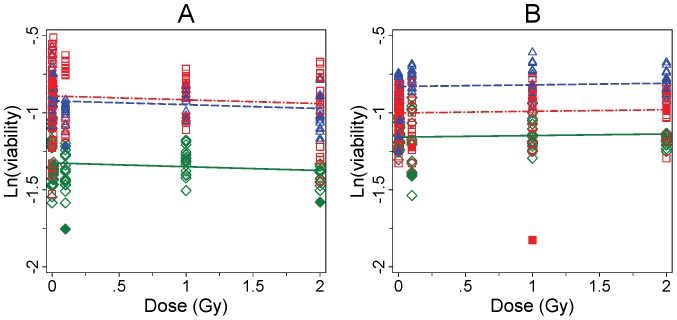
Cell viability in bystander cells. See Figure 4 legend for details.

The cell viability of *ATM* mutation carrier, A-T patient (biallelic *ATM* mutation) and wild type lymphoblastoid cell lines (LCLs) was analysed according to Pylkäs et al [12] using MTT cell viability test to see whether the increased CA yield affected the overall survival of the different cell lines. Figures 4 and 5 show cell viability data for directly irradiated and bystander cells, respectively. First of all, it is noteworthy that the baseline viability, i.e. survival of non-exposed control cells, varied between the cell lines (p<0.001). It is of interest that the viability of AT cells is greater than that of wild type cells or AT heterozygote. This may be due to intrinsic changes in the AT cell line that induces growth advantage. Regardless of the higher metabolic viability of these cells, the characteristic high baseline of CA frequency was observed. The viability of the three LCLs after direct irradiation showed clear differences in dose-dependent survival rates as demonstrated with the fitted models displaying significant discrepancies between the viability slopes in Figure 2 (p = 0.0084). At 44 hours (panel A) a significant decline in cell survival was seen in the *ATM* carrier and A-T LCLs with −0.23 (95% CI −0.33,−0.14) and −0.34 (95% CI −0.44,−0.25) in the logarithm of viability (ln(viability)) per Gy, respectively. In contrast, the ln(viability) of the WT LCL did not decrease significantly (−0.10 (95% CI −0.21,0.02) per Gy). As expected, the most radiosensitive cell line was the A-T LCL. As illustrated in lower panel in Figure 4, the effect of dose in the low dose range was similar in all cell lines indicating that 0.01 Gy or 0.1 Gy did not affect their viability (p = 0.18). At 63 hours post irradiation, the effect of dose was similar in all analyzed cell lines (Figure 4, panel B). The ln(viability) at 63 hours declined by −0.23 (95% −0.28,−0.18) per Gy. The cell viability assay was performed for the bystander cells at the same time points as the directly irradiated cells (Figure 5). The cell viability of the bystander cells was not affected by dose at 44 h (p = 0.20) or at 63 h (p = 0.35) in any of the analyzed cell lines. The data from all viability assay analyses are presented in Table S2.

## Discussion

Impact of low dose X-ray exposure on direct and bystander responses was analyzed by means of a co-culture system. Both low and high dose radiation-sensitivity was investigated using a previously characterized LCL from a breast cancer patient carrying an *ATM* mutation, an LCL from an A-T patient as well as a wild type LCL as a control.

The high baseline frequency of CAs is a recognized feature of the A-T cells and has previously been shown in A-T lymphocytes or LCLs in several investigations [10], [20]–[24] and was also observed in our study. At low doses (0.01 and 0.1 Gy), the chromosome or chromatid type aberrations were not significantly increased in relation to the controls for the carrier or for the A-T cell line and are generally in line with previous studies [10], [14]. However, there was a tendency, albeit statistically non-significant, of higher unstable chromosomal aberration frequency for the A-T cell line at 0.1 Gy. This result is consistent with results of Higurashi and Conen [20] who irradiated blood of three A-T children with 10 rad of γ-rays and performed analysis at first post-irradiation cell divisions. The authors observed that the frequency of both chromosome deletions and dicentrics and rings was increased in A-T cells compared to control cells although the increase was not statistically significant. Moreover, in line with our results on high-dose radiosensitivity of the *ATM* carrier LCL, Neubauer et al. [24] detected that a carrier can be distinguished from A-T and WT on the basis of the fraction of unstable CAs after X-irradiation of LCLs.

In cells exposed to direct irradiation, significant dose-dependent reduction in cell viability was detected in the *ATM* carrier and A-T LCLs when compared to the WT LCL. These results were expected because A-T cells are generally known to be extremely radiosensitive. This was also the case for the *ATM* carrier LCL. The same cell line was defined as highly sensitive cell line in a previous study [12]. The carrier LCL of the truncating 6903insA mutation was found to be indistinguishable from A-T cell lines in response to radiation. However, at the low dose range (up to 0.1 Gy) studied in our investigation, no reduction in cell viability was observed in any of the LCLs.

In the present study, bystander effect was not detected in the studied cell lines using cell viability and CA analysis as the end points at either low or high irradiation doses. However, the study design covered a relatively short time span after irradiation, and thus any bystander mediated delayed effects cannot be ruled out. Both unstable and chromatid-type CAs remained at baseline level in the bystander cells. In the A-T cells, the high intrinsic level of unstable CA sets a high background “noise” and thus prevents the identification of any subtle changes in the effect. In the literature, the number of papers dealing with bystander studies on A-T cells is very small and none of them concern low dose (≤0.1 Gy) irradiation or *ATM* carrier cells. Baskar et al [25] showed that after γ-irradiation, medium from *ATM*-deficient fibroblasts was less efficient in stimulating clonogenic potential of the bystander normal fibroblasts than the medium from the normal fetal lung fibroblasts. On the other hand, Burdak-Rothkamm et al. [26] demonstrated that clonogenic survival decreased in WT cells, but not in A-T bystander fibroblasts when filtered medium was derived from irradiated glioma cells, indicating the differential role of *ATM* in either targeted or non-targeted effects of radiation. Using a similar experimental co-culture design as applied in the present study, Yang et al. [27], [28] demonstrated reduced viability of bystander cells analyzed by the clonogenic assay after X-ray and Fe ion exposures. The reduction was dose-dependent below 0.5 Gy whereas no dose dependency from 0.5 Gy to 10 Gy was observed. On the other hand, no responses were induced in the bystander cells after medium transfer experiment from X-ray irradiated cells [29]. It has been suggested that specific experimental conditions may affect the existence of bystander response, such as culture conditions (medium, serum derived factors) [29]–[31]. The genetic background of the cell line may also explain the disparities [5].

In conclusion, low-dose hypersensitivity was not observed in the radiation sensitive *ATM* mutation carrier cell line, studied in a co-culture system studied with induced CA. However, at high dose exposure of 1 and 2 Gy, the *ATM* mutation carrier cell line showed significant increase in CA, demonstrating intermediate response of radiation sensitivity. Cell viability tests corroborated the findings. No indication of medium-borne bystander effect was detected in any of the cell lines or at any doses in the co-culture setting applied. Finally, typical features for the A-T patient cell line were displayed: high baseline in addition to dose-dependent increase of unstable chromosome-type CA, as well as loss of cell viability.

## Supporting Information

Table S1 **Supporting data on chromosomal aberration results.**
(XLSX)

Table S2 **Supporting data on viability assay results.**
(XLSX)
